# Supplementary material for: Visualizing the Functional Dynamics of P-Glycoprotein and Its Modulation by Elacridar via High-Speed Atomic Force Microscopy
Source: Int J Mol Sci. 2025 Dec 29;27(1):356. doi: 10.3390/ijms27010356 (PMC12785683; doi:10.3390/ijms27010356)
Supplement: Supplementary file 1 [file ijms-27-00356-s001.zip › Supplymental_figures_table.pdf]

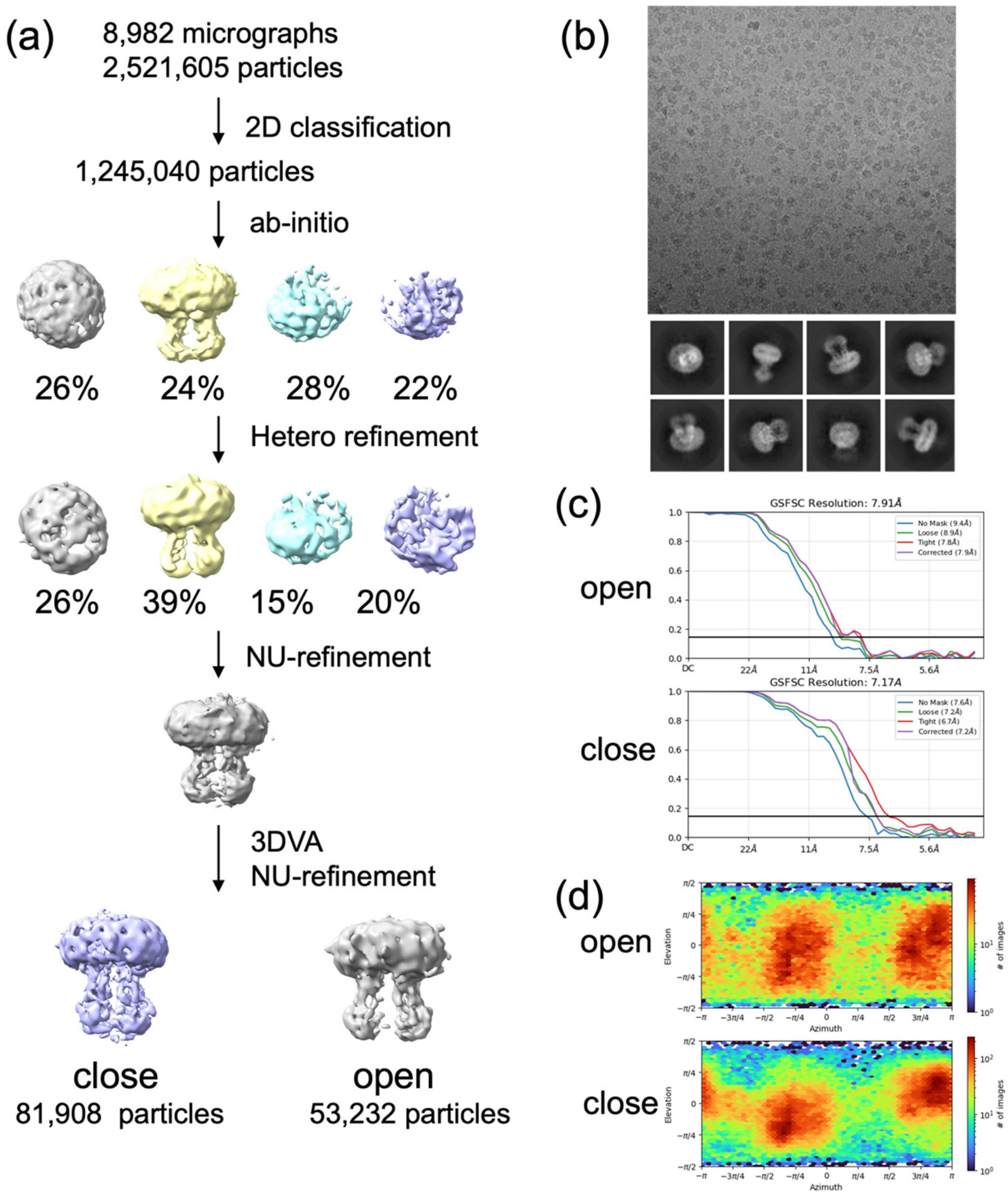

Figure S1. cryo-EM analysis information.

(a) Workflow of EM structure determination.

(b) Representative micrograph, alongside 2D class averages.

(c) Fourier shell correlation (FSC) curves showing resolution estimates, determined at a threshold of 0.143.

(d) Angular distribution calculated for particle projections.

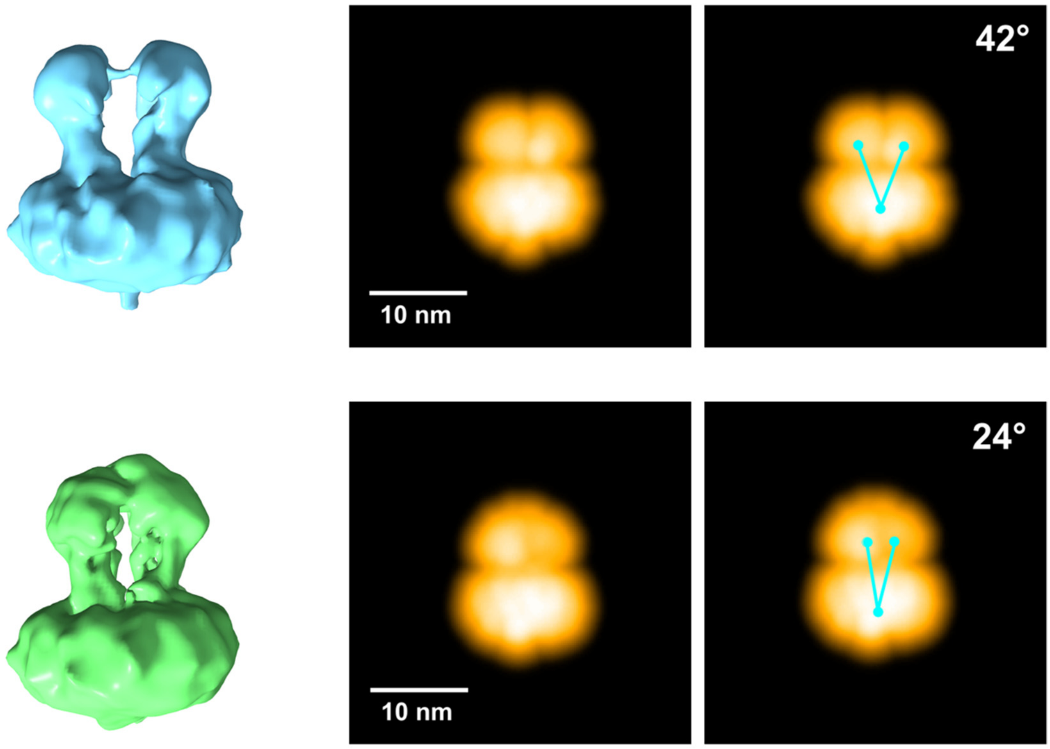

Figure S2. Simulated AFM images generated from cryo-EM maps.

Simulated AFM images were generated based on the electron density maps obtained by cryo-electron microscopy, using a probe tip radius of 1.0 nm and a cone angle of 20°. The upper and lower panels depict the open and closed NBD conformations, respectively. The three light blue dots indicate the points used for the angle measurement.

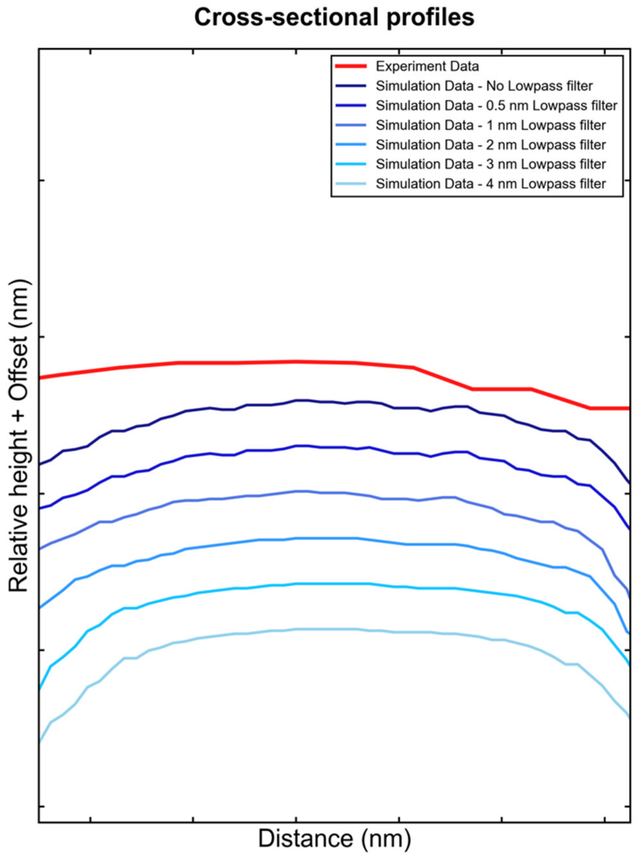

Figure S3. Cross-sectional profiles of the AFM experiment image and simulated images with different cut-off filters.

Cross-sectional profiles were taken for the AFM experiment image and simulated AFM images with applying different lowpass filters of P-gp-ND. To make the graph easier to read, we are displaying it with an offset applied to the Y-axis.

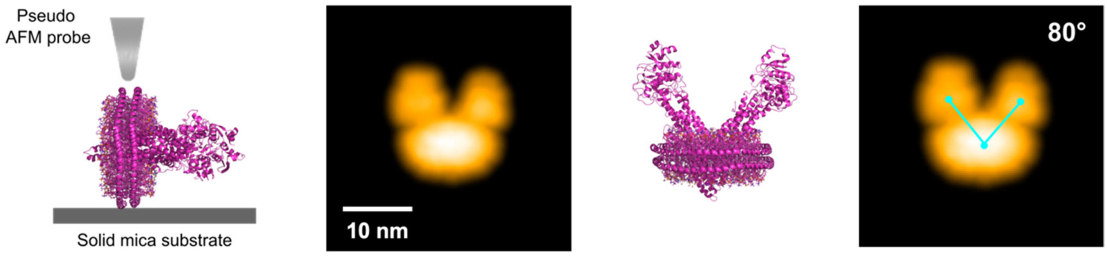

Figure S4. Structural dynamics simulation by NMA and generation of the corresponding simulated AFM image.

Normal Mode Analysis (NMA) was applied to the apo state structure (PDB: 7OTI) to simulate its conformational dynamics and reproduce the structure observed by HS-AFM. A simulated AFM image was then generated from a conformation generated via NMA using a tip radius of 1 nm and a cone angle of 20°, consistent with the parameters used for the other simulations. The three light blue dots indicate the points used for the angle measurement.

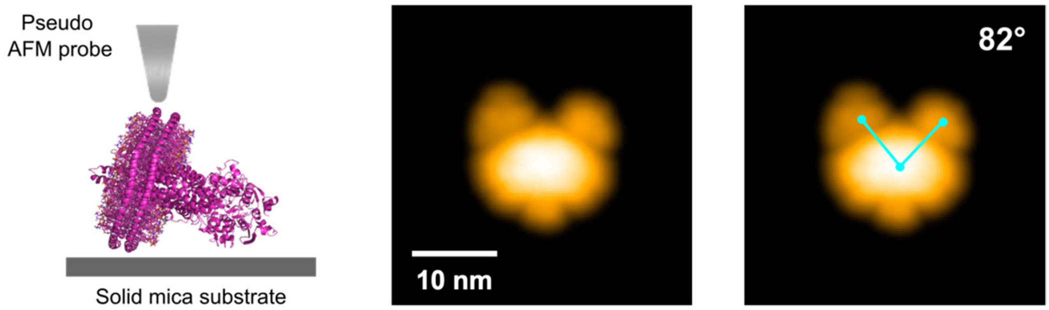

Figure S5. Simulated AFM image derived from the NMA structure tilted relative to the substrate. A simulated AFM image was generated from the NMA-derived conformation, tilted relative to the substrate, using consistent parameters (tip radius of 1 nm and cone angle of 20°). The three light blue markers designate the specific measurement points used to calculate the opening angle.

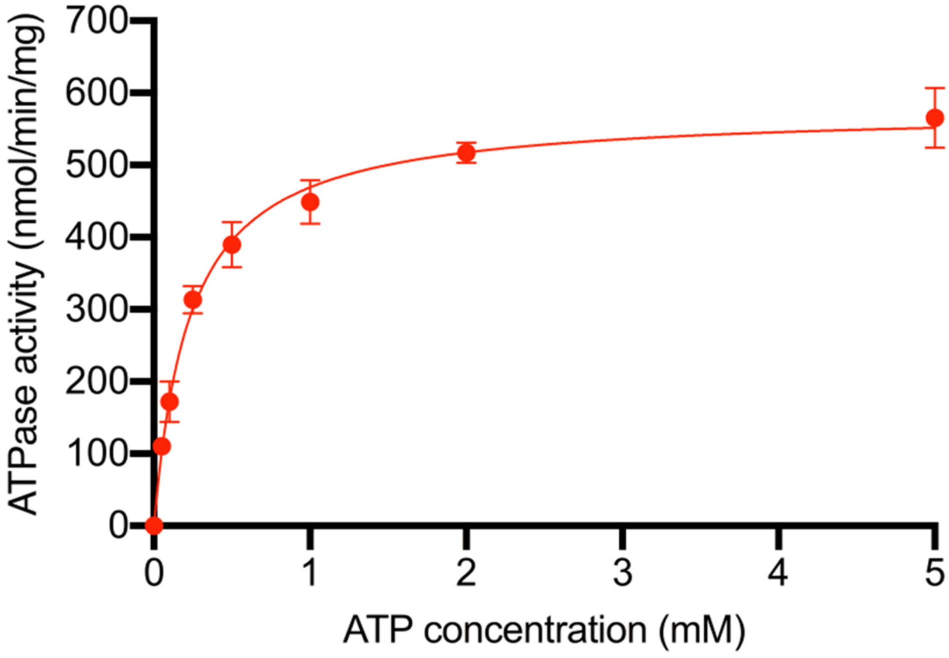

Figure S6. Michaelis-Menten kinetics of P-gp-ND ATPase activity.

ATP hydrolysis rates of P-gp-ND were measured over increasing ATP concentrations using an ATP-regenerating system (see Materials and Methods). The data points represent the mean ± standard deviation of three independent experiments.

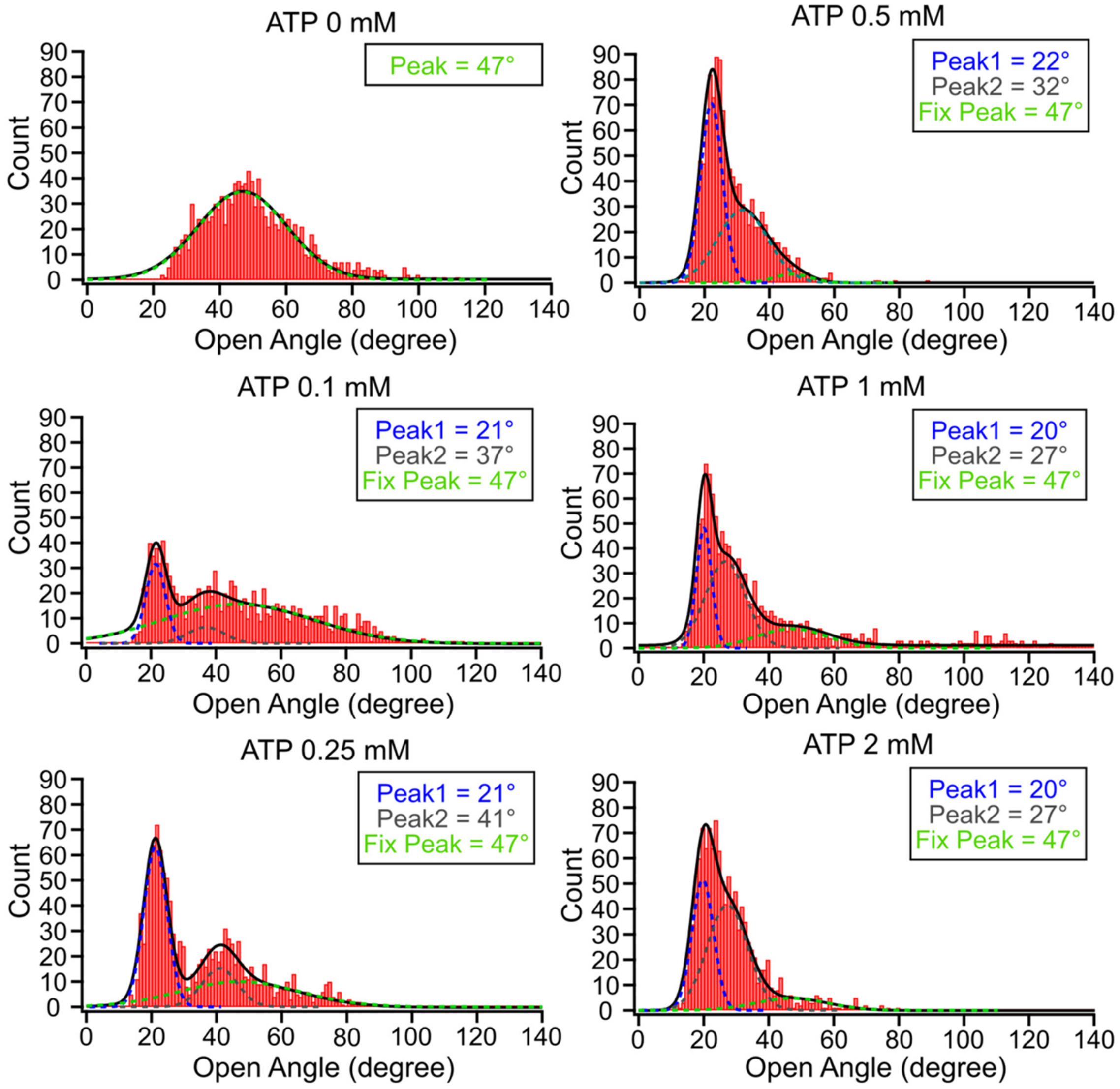

Figure S7. Histogram of NBD opening angles at various ATP concentrations.

Histograms of NBD opening angles in the presence of ATP at the indicated concentrations. Each histogram was constructed from the analysis of 1,200 frames.

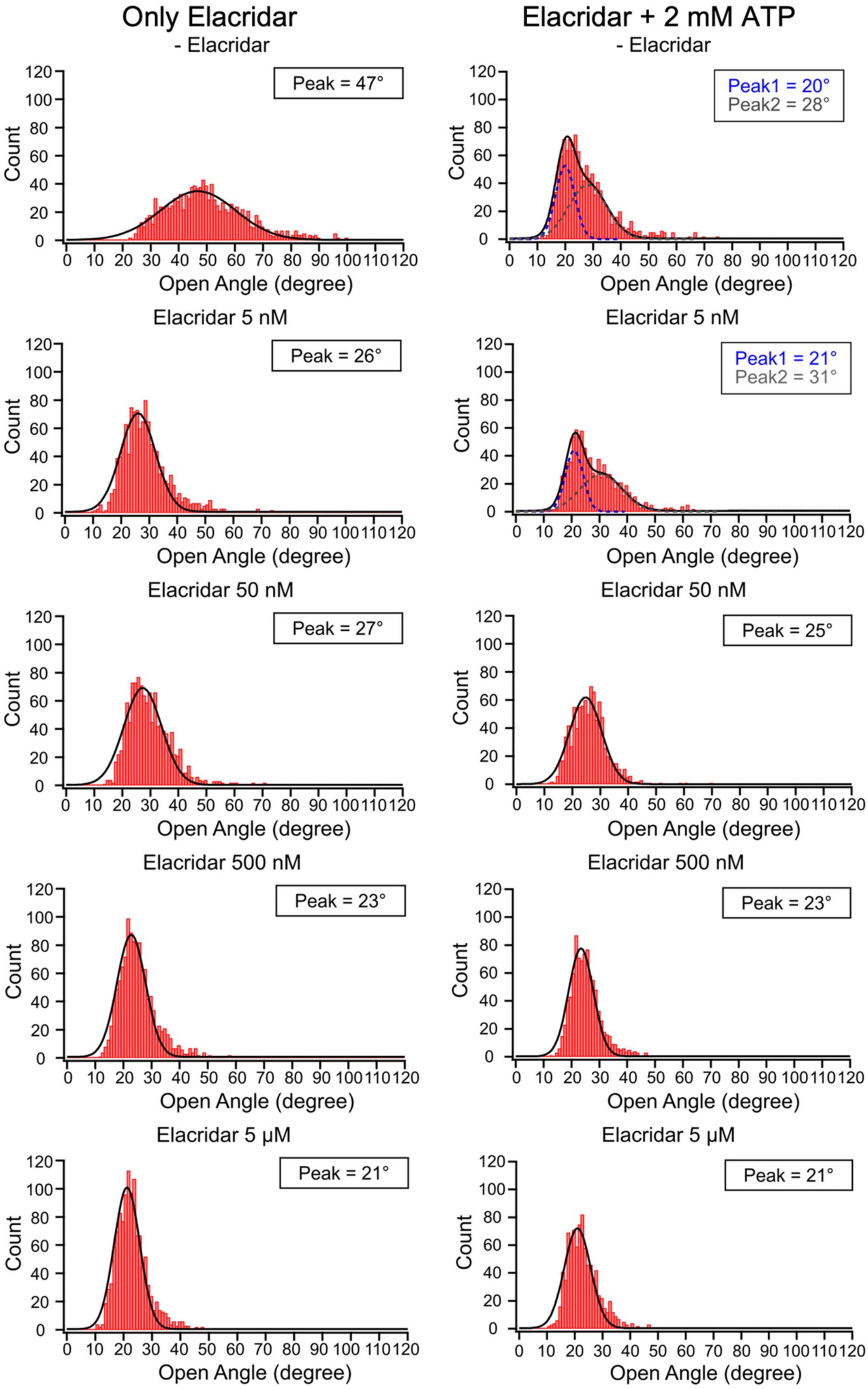

Only Elacridar
Elacridar + 2 mM ATP
- Elacridar
Peak = 47°
Peak1 = 20°
Peak2 = 28°
Elacridar 5 nM
Peak = 26°
Peak1 = 21°
Peak2 = 31°
Elacridar 50 nM
Peak = 27°
Peak = 25°
Elacridar 500 nM
Peak = 23°
Peak = 23°
Elacridar 5 μM
Peak = 21°
Peak = 21°
Count
Open Angle (degree)

Figure S8. Histogram of NBD opening angles in the presence of either elacridar alone or Elacridar and 2 mM ATP.

Histograms of NBD opening angles in the absence and presence of ATP at the indicated Elacridar concentrations. Each histogram of Elacridar alone condition was constructed from 1,200 frames. The histograms were constructed from 1,200 frames for conditions with either Elacridar or 2 mM ATP alone, and from 900 frames for conditions where both were present.

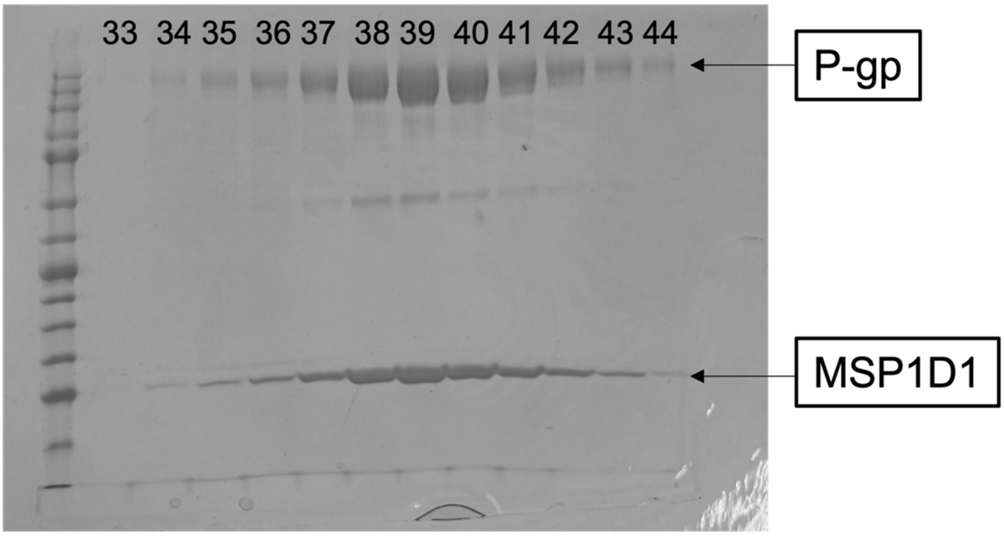

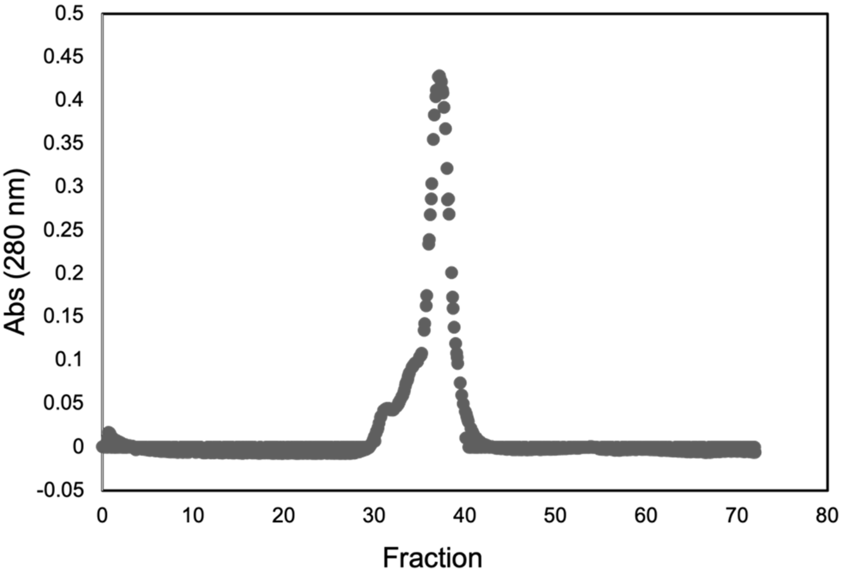

Figure S9. Size exclusion chromatography (SEC) elution profile and SDS-PAGE result for P-gp-ND purification.

Upper panel shows the SDS-PAGE result of P-gp reconstituted with Nanodisc. Lower represents the SEC elution profile.

Data collection	Open state	Close state
EMDB	EMD-66042	EMD-66043
Microscope	Titan Krios (Thermo Fisher Scientific)	
Detector	Falcon 4i	
Magnification	105000	105000
Voltage (kV)	300	300
Electron exposure (e^-/Å^2)	50	50
Defocus range (μm)	0.8-2.0	0.8-2.0
Pixel size (Å)	0.75	0.75
Symmetry	C1	C1
Micrographs	8982	8982
Initial particle images	2,521,605	2,521,605
Final particle images	53,232	81,908
Map resolution (Å)	7.89	6.76
FSC threshold	0.143	0.143

Table S1. cryo-EM data collection.
